# Association between Plasma PFOA and PFOS Levels and Total Cholesterol in a Middle-Aged Danish Population

**DOI:** 10.1371/journal.pone.0056969

**Published:** 2013-02-18

**Authors:** Kirsten T. Eriksen, Ole Raaschou-Nielsen, Joseph K. McLaughlin, Loren Lipworth, Anne Tjønneland, Kim Overvad, Mette Sørensen

**Affiliations:** 1 Danish Cancer Society Research Center, Copenhagen, Denmark; 2 International Epidemiology Institute, Rockville, Maryland, United States of America; 3 Department of Epidemiology, School of Public Health, Aarhus University, Aarhus, Denmark; Indian Institute of Toxicology Research, India

## Abstract

Perfluorooctanoate (PFOA) and perfluorooctane sulfonate (PFOS) are used in a variety of consumer products and have been detected worldwide in human blood. Recent studies mainly of highly exposed populations have indicated that PFOA and PFOS may affect serum cholesterol levels, but the magnitude of the effect may be inconsistent across exposure levels. The aim of the present cross-sectional study was to investigate the association between plasma PFOA and PFOS and total cholesterol in a general, middle-aged Danish population. The study population comprised 753 individuals (663 men and 90 women), 50–65 years of age, nested within a Danish cohort of 57,053 participants. Blood samples were taken from all cohort members at enrolment (1993–1997) and stored in a biobank at -150°C. Plasma levels of PFOA and PFOS and serum levels of total cholesterol were measured. The associations between plasma PFOA and PFOS levels and total cholesterol levels were analysed by generalized linear models, both crude and adjusted for potential confounders. We observed statistically significant positive associations between both perfluorinated compounds and total cholesterol, e.g. a 4.4 [95% CI  =  1.1–7.8] higher concentration of total cholesterol (mg/dL) per interquartile range of PFOA plasma level. Sex and prevalent diabetes appeared to modify the association between PFOA and PFOS, respectively, and cholesterol. In conclusion, this study indicated positive associations between plasma PFOA and PFOS levels and total cholesterol in a middle-aged Danish population, although whether the observed pattern of results reflects a causal association is unclear.

## Introduction

Perfluorooctanoate (PFOA) and perfluorooctane sulfonate (PFOS) are synthetically produced perfluorinated chemicals (PFCs) known for their antiwetting and surfactant properties. They are widely used in commercial applications such as nonstick cookware, waterproof, breathable textiles and protective coatings for paper, food packing materials, and carpets [1], [2]. PFOA and PFOS are resistant to metabolic and environmental degradation, are bioaccumulative, and have been found in human blood and tissue samples from occupationally exposed workers and in the general population worldwide [3]–[6].

PFOA and PFOS have in some studies been positively associated with serum lipid levels such as cholesterol, which may increase the risk of cardiovascular diseases. Most human studies investigating the association between PFCs and cholesterol have been carried out in populations with high PFC levels in blood, such as occupationally exposed workers or highly exposed communities (reviewed in [7]). Associations between PFOA and cholesterol have been investigated in six occupational studies [8]–[13], all of which indicated positive associations, although the magnitude of the association was inconsistent across exposure levels [7]. Positive associations were also found in two studies of highly exposed communities [14], [15], whereas no association was found in a third study [16]. A recent US general population study based on data from 860 individuals observed positive associations between PFOA, PFOS and perfluoroctanoic acid and total cholesterol and non-HDL-cholesterol. In contrast, the opposite association was found for the less studied perfluorinated compound perfluorohexansulfonate [17]. In the same study, no consistent associations were observed between HDL-cholesterol and any of the studied PFCs and between LDL-cholesterol and PFOA and PFHxS. Thus, the epidemiologic evidence of associations between PFOA and cholesterol in human is not consistent with what has been observed in animal feeding studies, in which PFOA has shown a negative relationship with cholesterol concentrations in rodents and no relationship with cholesterol in non-human primates, while PFOS has shown a negative relationship with cholesterol concentrations in rodents and monkeys [18].

The aim of the present study was to investigate the association between plasma PFOA and PFOS levels and total cholesterol concentrations in a general, middle-aged Danish population.

## Materials and Methods

### Ethics Statement

The study was approved by the regional research ethic committee for Copenhagen and Frederiksberg. Written informed consent was obtained from all study participants. The study was carried out without contact to the cohort members or their families. Anonymity of participants was retained by strict data management.

### Study Population

Between 1993 and 1997, 57,053 individuals (27,178 men and 29,875 women) 50–65 years of age, born in Denmark and with no previous cancer diagnosis, were enrolled in the prospective Danish Diet, Cancer and Health (DCH) cohort [19]. Information on lifestyle factors, including dietary, drinking and smoking habits, as well as medical and reproductive history was obtained at enrolment. Blood samples were drawn from each participant at enrolment and stored at -150°C. In a previous study [20], PFOA and PFOS plasma levels were measured among 1240 individuals from the DCH cohort diagnosed with prostate, bladder, pancreatic, or liver cancer after enrolment and for a randomly selected comparison group of 772 individuals from the same cohort with the same male-to-female ratio as the cancer cases. The present analysis was based on the comparison group of the described study; among the 772 individuals we excluded 17 who reported taking medication for high levels of cholesterol and 2 with no information on total cholesterol levels, resulting in a study population of 753 individuals (663 men and 90 women).

### Measurement of total cholesterol and plasma PFOA and PFOS levels

30 mL non-fasting blood samples were drawn from each participant at enrolment, spun and divided into 6×1 mL of plasma, 4×1 mL of serum and 2×1 mL of erythrocytes and 2×1 mL of buffy coat and stored at -150°C. For the present study we used the PFOA and PFOS plasma levels that were measured in the previous study [20] and we used the total cholesterol levels that were determined on the day of enrolment at the time of blood sampling.

Concentrations of PFOA and PFOS in plasma were determined using high performance liquid chromatography/tandem mass spectrometry at the 3M Toxicology Laboratory, using the methods described by Ehresman et al. [21]. The lower limit of quantification (*L*) for the method was 1.0 ng/mL for both PFCs. In our analysis, PFOS was measured above *L* in all samples and two PFOA values were below the *L* and were assigned the value 0.71 ng/mL, according to the formula *L*/√2 for replacement of non-detectable values [22]. As a quality control measure, 50 blind samples were selected for repeated analysis of PFOA and PFOS concentrations to ensure the accuracy and reliability of the data. The 3M Toxicology Laboratory was blinded to this series of parallel measurements. Mean coefficients of variation were low (5.9% for PFOA and 1.8% for PFOS).

Non-fasting total cholesterol (mmol/L) was determined in whole blood using a Lipotrend® C device with Lipotrend test strips (Boehringer Mannheim). Lipotrend® C is a reflectance photometer for the determination of total cholesterol from capillary blood or plasma in the range of 100–500 mg/dl (2.6–13.0 mmol/L). The sample was applied onto the test strip by means of a plastic capillary. Cholesterol determination was automatically begun upon correct insertion of the test strip into the instrument. The optical system measures the color intensity of the test strip’s reaction zone at two different times, and the difference is converted into the cholesterol concentration using the Lipotrend® C software and shown in the display. For our analyses, the SI unit (mmol/L) was converted to mg/dL by multiplying by 38.67.

### Statistical analyses

The association between PFOA and PFOS plasma levels and total cholesterol were analyzed by generalized linear models. Linearity of the associations between PFCs and cholesterol was verified by linear splines, and the primary analyses were conducted using PFOA and PFOS as continuous variables. In addition, for visualization of the results we investigated cholesterol levels in relation to categorization of PFOA and PFOS concentrations into eight groups, with approximately 100 persons in each exposure group, using the lowest exposure group as reference. Regression analyses were performed (with adjustment for sex only) and adjusted regression analyses were performed with adjustment for sex, age, years of school attendance (<8, 8–10, or >10 years), body mass index (BMI; kg/m^2^), smoking status (present, former, or never smoker), alcohol intake (g/day), egg intake (g/day), animal fat intake (g/day) and physical activity (MET score; hours/week). Some of the adjustment variables were selected based on what is previously shown to be associated with PFC levels in this population [23], such as BMI and alcohol (inversely), sex, smoking status and egg intake. Risk factors for high cholesterol level include diet high in saturated animal fats and low physical activity, therefore these factors were taken into account. Also, we investigated whether sex, BMI and prevalent diabetes modified the association between PFOA/PFOS and cholesterol. The analyses were conducted using the PROC GLM procedure of SAS 9.1 (SAS Institute Inc., Cary, NC).

## Results

The 753 study participants had a mean serum cholesterol level of 232 mg/dL. Women had significantly higher concentrations of cholesterol than men with a difference of 18.5 mg/dL (95% CI: 9.6–27.3). Mean plasma PFOA and PFOS levels were 7.1 ng/mL and 36.1 ng/mL, respectively. Men had significantly higher levels of both compounds with a difference of 1.5 ng/mL (95% CI: 0.9–2.2) for PFOA and a difference of 6.1 ng/mL (95% CI: 3.0–9.2) for PFOS.

Linearity of the associations between PFOA and PFOS and total cholesterol was verified in spline analyses showing that the associations did not deviate significantly from linearity (the p-values were 0.58 and 0.22 in the spline analysis for PFOA and PFOS, respectively).

Table 1 shows the association between PFOA and PFOS and total cholesterol concentrations. We found statistically significant positive associations between both perfluorinated compounds and the level of total cholesterol in the adjusted models (sex and fully adjusted), e.g. a 4.4 [95% CI  =  1.1–7.8] higher concentration of total cholesterol (mg/dL) per interquartile range of plasma PFOA in the fully adjusted model. There were only small differences in the estimates for the models adjusted for sex as compared with the fully adjusted models. In the crude models the associations where positive but insignificant: a 2.9 [95% CI  =  -0.3; 6.3] and a 2.6 [95% CI  =  -1.1; 6.2] higher concentration of cholesterol per interquartile range of PFOA and PFOS, respectively.

**Table 1:** Differences in total cholesterol level (mg/dL) per interquartile range of plasma PFOA and PFOS.

		PFOA						PFOS					
		Adjusted for sex			Adjusted for sex, education, age and lifestyle covariates a			Adjusted for sex			Adjusted for sex, education, age and lifestyle covariates a		
	N	Effect (95%CI)	*p*	*p* b	Effect (95%CI)	*p*	*p* b	Effect (95%CI)	*p*	*p* b	Effect (95%CI)	*p*	*p* b
Total population	753	**4.1 (0.8;7.4)**	**0.01**		**4.4 (1.1;7.8)**	**0.01**		**3.7 (0.1;7.3)**	**0.04**		**4.6 (0.8;8.5)**	**0.02**	
Effect modification													
Sex				**0.04**			**0.04**			0.17			0.17
Women	90	15.5 (4.0;27.2)			15.8 (4.2;27.4)			11.7 (–0.2;23.6)			12.5 (0.5;24.5)		
Men	663	3.1 (–0.3;6.5)			3.4 (0.1;6.9)			2.9 (–0.9;6.7)			3.7 (–0.3;7.8)		
Diabetes				0.57			0.52			**0.04**			**0.03**
Yes	22	12.0 (–15.5;39.5)			13.4(–14.1;40.8)			29.2 (4.0;54.4)			32.0 (6.8;57.2)		
No	729	4.0 (0.6;7.3)			4.3 (0.9;7.7)			3.0 (–0.7;6.7)			4.0 (0.2;7.9)		
BMI				0.51			0.40			0.64			0.62
<25	261	5.6 (0.1;11.1)			6.3 (0.7;11.9)			4.9 (–0.5;10.2)			5.8 (0.2;11.3)		
>25	492	3.4 (–0.7;7.4)			3.4 (–0.8;7.5)			3.2 (–1.7;8.0)			3.9 (–1.1;9.0)		

a Lifestyle covariates: BMI, smoking status, intake of alcohol, egg, and animal fat and physical activity.

b *p* value for effect modification.

**Figure 1:**
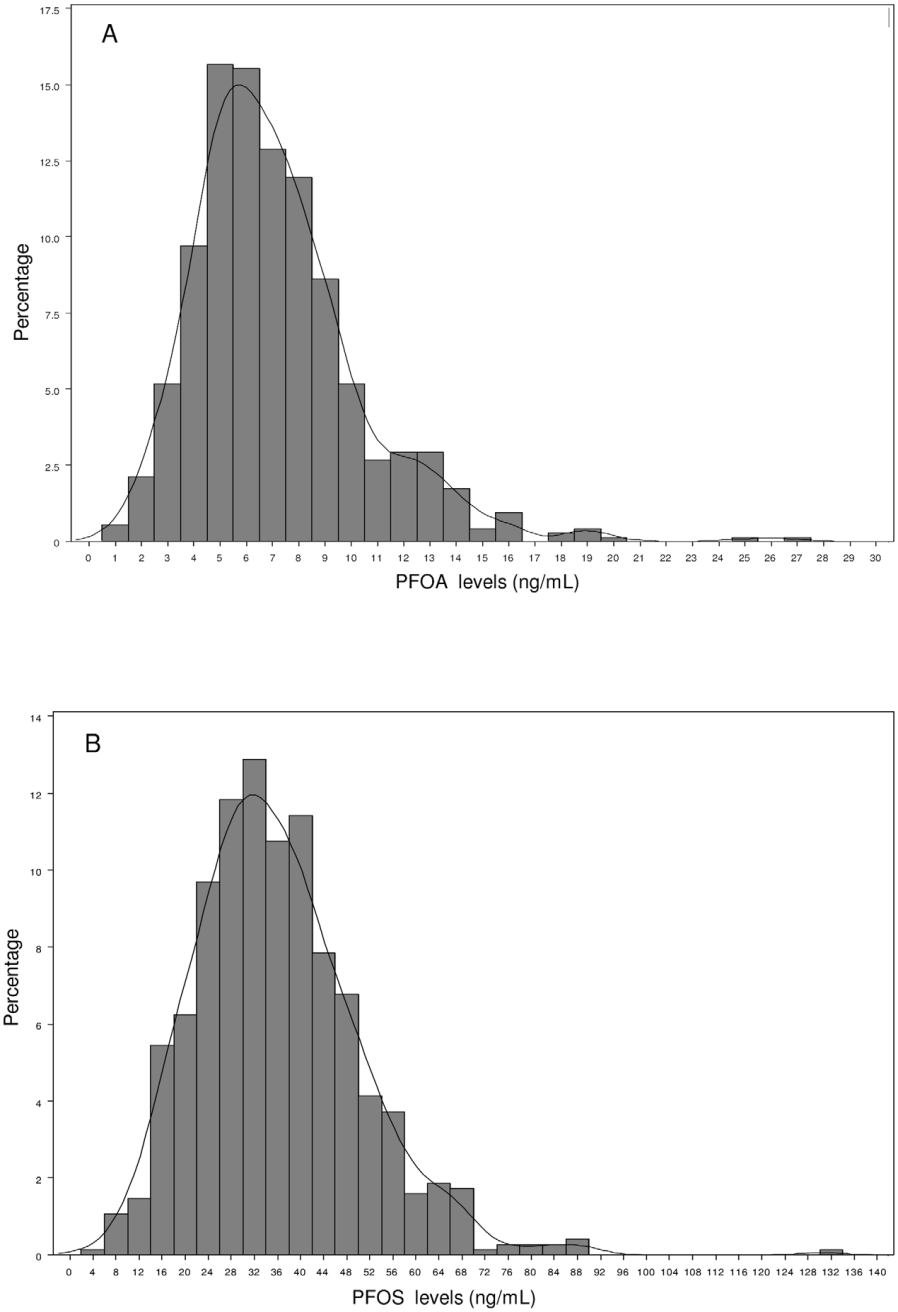
Distributions of plasma PFOA and PFOS levels. (A) Distribution of plasma PFOA levels (ng/mL) of the study population. (B) Distribution of plasma PFOS levels (ng/mL) of the study population.

The distributions of PFOA and PFOS values are shown in Figure 1A and 1B. Exclusion of outliers for PFOA from the data set gave similar results as those reported in Table 1 (4.2 [95% CI  =  0.7–7.7] in the sex-adjusted model), whereas for PFOS the associations only slightly increased (4.5 [95% CI  =  4.5 (0.8–8.3] in the sex-adjusted model).

Sex significantly modified the association between PFOA and cholesterol, with a stronger association among women, and prevalent diabetes significantly modified the association between PFOS and cholesterol, with a stronger association among diabetics. BMI did not modify the associations (Table 1).

**Figure 2:**
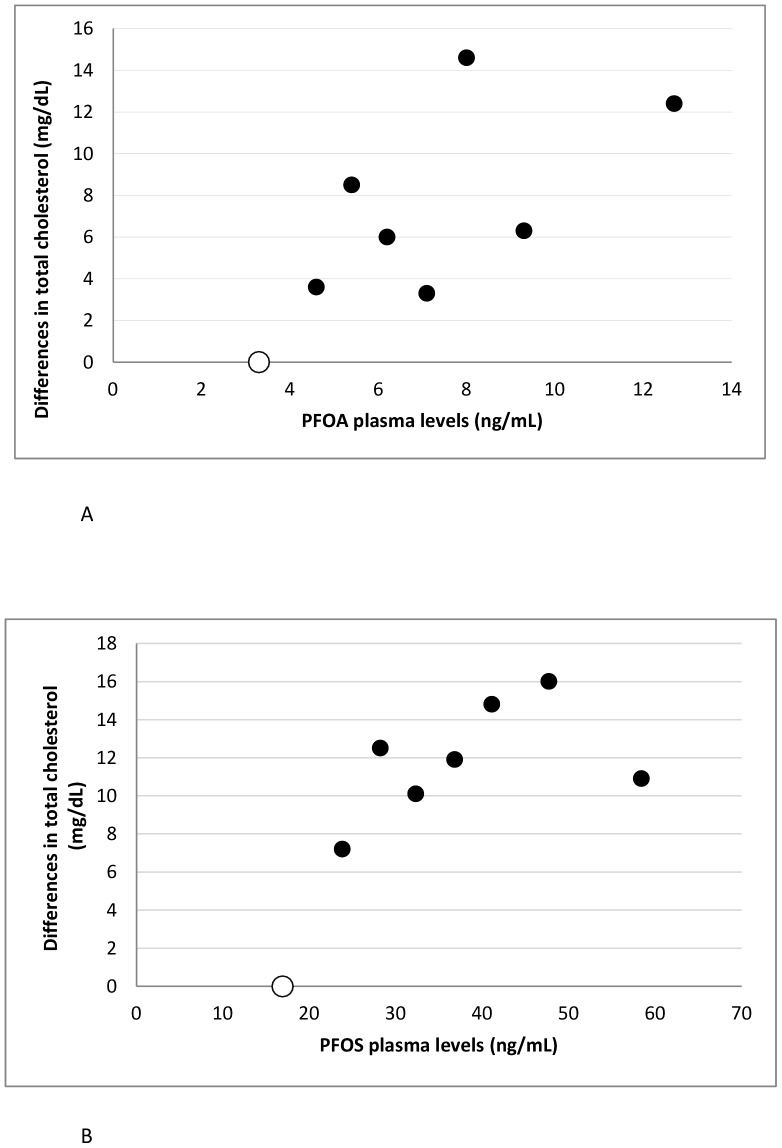
Associations between plasma PFOA and PFOS levels and total cholesterol. The Figure illustrates differences in total cholesterol levels according to eight exposure groups of (A) PFOA and (B) PFOS. The lowest exposure groups are used as reference (open dots). The dots are placed at the median of the eight exposure categories. Models are adjusted (sex, age, years of school attendance, body mass index, smoking status, alcohol intake, egg intake, animal fat intake and physical activity).

Figure 2 shows differences in total cholesterol levels according to eight exposure groups of PFOA levels (Figure 2A) and PFOS levels (Figure 2B), where the lowest exposure group is used as reference group (adjusted associations presented). The level of total cholesterol appears to be higher with higher PFOA plasma levels with a borderline significant p value for trend. Similar tendencies are seen for PFOS although the association seems to level slightly off at about 40 ng/mL PFOS.

## Discussion

Risk factors for high cholesterol level include diet high in saturated animal fats, low physical activity, and a family history of the condition, but increasing evidence indicates that environmental chemicals may also contribute. In this study, we found positive associations between plasma PFOA and PFOS levels and total cholesterol in a general, middle-aged Danish population. Total cholesterol levels were about 4 mg/dL higher per IQR of plasma PFOA and PFOS levels, and the association seemed to follow a dose-response pattern.

The mechanism by which perfluorinated compounds are related to cholesterol in humans is unknown, but the finding of positive associations between perfluorinated compounds and cholesterol levels contradicts what would be expected from animal studies. In animals, PFOA is a strong peroxisome-proliferator-activated receptor-alpha in the liver and this proliferation has been shown to alter lipid metabolism with decreases, not increases, serum lipids [24]. This peroxisome proliferation is much less apparent in humans [25] and may clarify the discrepancy between human and animal studies on the associations between perfluorinated compounds and cholesterol levels. When PFCs enter the human body, the compounds accumulate in blood (bound to serum proteins), kidney and liver [24]. Cholesterol is produced in part by the liver where PFCs may induce production. Cholesterol is excreted by the liver via the bile into the digestive tract and some is reabsorbed into the bloodstream. Due to our cross-sectional design we cannot determine any temporal relation between total cholesterol and PFCs. Alternative explanations for the association could be that the study group is exposed to other undefined exposures, which may correlate both with higher cholesterol level and with higher maintenance of PFOA and PFOS in the blood. Also, the observed association could be due to dietary habits associated with exposure to PFOA and PFOS and with levels of cholesterol. However, adjustment for intake of eggs, which we have previously found to be associated with PFOS [23], lead to only minor changes in the estimates. It should be acknowledged, however, that this statistical adjustment was based on self-reported dietary data taken solely at enrolment. The mechanisms by which PFCs provoke their effects in humans are not well known. The effect of PFC exposure could be affected by genetic differences causing some individuals to be more susceptibility to these compounds and their potential biological effects. However, PFCs are chemically stabilized by strong covalent C–F bonds and are considered to be metabolically inert. To the best of our knowledge no studies have show inter-individually differences in response to PFC exposure due to genetic differences amd we believe that the possibility of individually differences at least in respect to polymorphisms of metabolic genes having affected the results of our study is limited. We also found that diabetes, which may stimulate cholesterol synthesis, and sex modified the association between PFOA and PFOS and cholesterol, suggesting differences in susceptibility to these compounds. In should be stated that the analyses on diabetes were only based on 22 diabetics, so these results should be interpreted with caution.

PFOA and PFOS are considered sensitive and accurate biomarkers of internal exposure to these substances [1]. In our study, non-differential misclassification may have occurred when using a single measurement of the plasma levels of PFOA and PFOS for each individual which may not reflect the plasma levels at other times. However, since half-lives of PFOA and PFOS have been reported to be 4 and 5 years, respectively [26], one measurement may represent a reasonably stable internal dose. Other limitations include the cross-sectional design with no repeated measurement of total serum cholesterol. Also, we did not have information on serum levels of LDL-cholesterol, which is the optimal substance for assessing individual risk of cardiovascular events. However, since there is a high correlation between total cholesterol and LDL-cholesterol, and since LDL-cholesterol represents around 70% of total cholesterol, total cholesterol levels can be used as a predictor of LDL-cholesterol and thus risk of cardiovascular diseases.

Our study is one of the first to show a positive association between PFC levels and cholesterol levels in a low-level exposed general population, which is supported by previous studies of mainly highly exposed populations [8]–[17], [27]. From a health perspective point of view it is, however, still unclear whether these minor differences in total cholesterol is important alone.

In conclusion, this study indicated small but significant associations between plasma PFOA and PFOS levels and total cholesterol in a middle-aged, general Danish population.
